# Multiple Occult Fractures at Hemodialysis Initiation in a Chronic Kidney Disease Patient: Diagnostic Value of MRI and Potential Role of Zinc Deficiency

**DOI:** 10.7759/cureus.99672

**Published:** 2025-12-19

**Authors:** Ryoichi Nakazawa, Akira Onozaki, Takashi Uchino, Nakanobu Azuma, Kenji Endo

**Affiliations:** 1 Department of Nephrology, Tokatsu-Clinic Hospital, Matsudo, JPN; 2 Department of Surgery, Tokatsu-Clinic Hospital, Matsudo, JPN; 3 Department of Orthopedic Surgery, Tokyo Medical University, Tokyo, JPN

**Keywords:** maintenance hemodialysis, muskuloskeletal mri, occult fracture, osteoporosis and kidney disease, zinc supplementation

## Abstract

We report the case of a 55-year-old man with end-stage kidney disease (ESKD) who developed multiple occult fractures within two months of hemodialysis initiation. Magnetic resonance imaging (MRI), but not radiography or computed tomography (CT), identified fractures in the calcanei, metatarsals, and pelvis. Laboratory findings revealed secondary hyperparathyroidism and hypozincemia. Treatment with calcimimetics, active vitamin D analogues, and zinc supplementation led to symptom resolution over 17 months. This case emphasizes the value of early MRI for unexplained pain at dialysis initiation and suggests that zinc deficiency may contribute to fracture risk in chronic kidney disease-mineral and bone disorder (CKD-MBD).

## Introduction

A heterogeneous clinical course characterizes chronic kidney disease (CKD); many patients experience a slowly progressive decline in kidney function, and a significant proportion progresses to end-stage kidney disease (ESKD), sometimes decades after the initial presentation. IgA nephropathy, the most common primary glomerular disease worldwide, is one such cause of CKD. A long-term observational study reported a 30-year renal survival rate of approximately 50.3% in IgA nephropathy, underscoring the chronic yet unpredictable nature of the disease [1].

CKD-mineral and bone disorder (MBD) is a major systemic complication of CKD, encompassing biochemical abnormalities, skeletal pathology, and vascular calcification [2]. Among these, skeletal manifestations, particularly fragility fractures, are a leading cause of morbidity and reduced quality of life. Fractures in CKD are frequently “occult,” lacking overt clinical symptoms due to neuropathy or altered pain perception, and remaining undetected without targeted imaging such as magnetic resonance imaging (MRI). Delayed recognition may lead to cumulative skeletal damage and increased mortality.

Although bone disease in patients undergoing dialysis has been extensively studied, the occurrence and clinical relevance of occult fractures at or near dialysis initiation remain insufficiently recognized. Moreover, early postdialysis fractures are often misattributed to the effects of uremia or immobility, potentially delaying appropriate management.

Here, we describe a rare and illustrative case of multiple occult fractures detected by MRI shortly after hemodialysis initiation in a patient with ESKD secondary to presumed IgA nephropathy. This case highlights the importance of early skeletal assessment during the transition to dialysis. It underscores the potential role of micronutrient deficiencies, such as zinc deficiency, in impaired bone quality by inhibiting osteoblastic bone formation and osteoclastic bone resorption and increasing fracture risk in CKD-MBD.

## Case presentation

A 55-year-old man was referred to our hospital for the initiation of maintenance hemodialysis due to ESKD. His renal history extended over three decades; proteinuria was incidentally detected during a workplace health screening when the patient was 25 years old, and a renal biopsy was not performed. At 41 years of age, laboratory evaluation revealed a serum creatinine level of 3.0 mg/dL, proteinuria of 67 mg/dL, and microscopic hematuria, leading to a diagnosis of CKD secondary to presumed IgA nephropathy. Conservative management with dietary and pharmacological interventions was then initiated.

At approximately 50 years of age, alfacalcidol was initiated for CKD-MBD treatment, and transient zinc supplementation was administered for hypozincemia (serum zinc 48-64 µg/dL) at 53 years of age. Two months after starting hemodialysis, the patient reported progressive pain and swelling in the left ankle and pain on the right foot dorsum without a history of trauma. Clinical evaluation suggested left Achilles tendinopathy and right extensor digitorum longus tenosynovitis, for which conservative treatment was initiated; however, the symptoms persisted.

**Figure 1:**
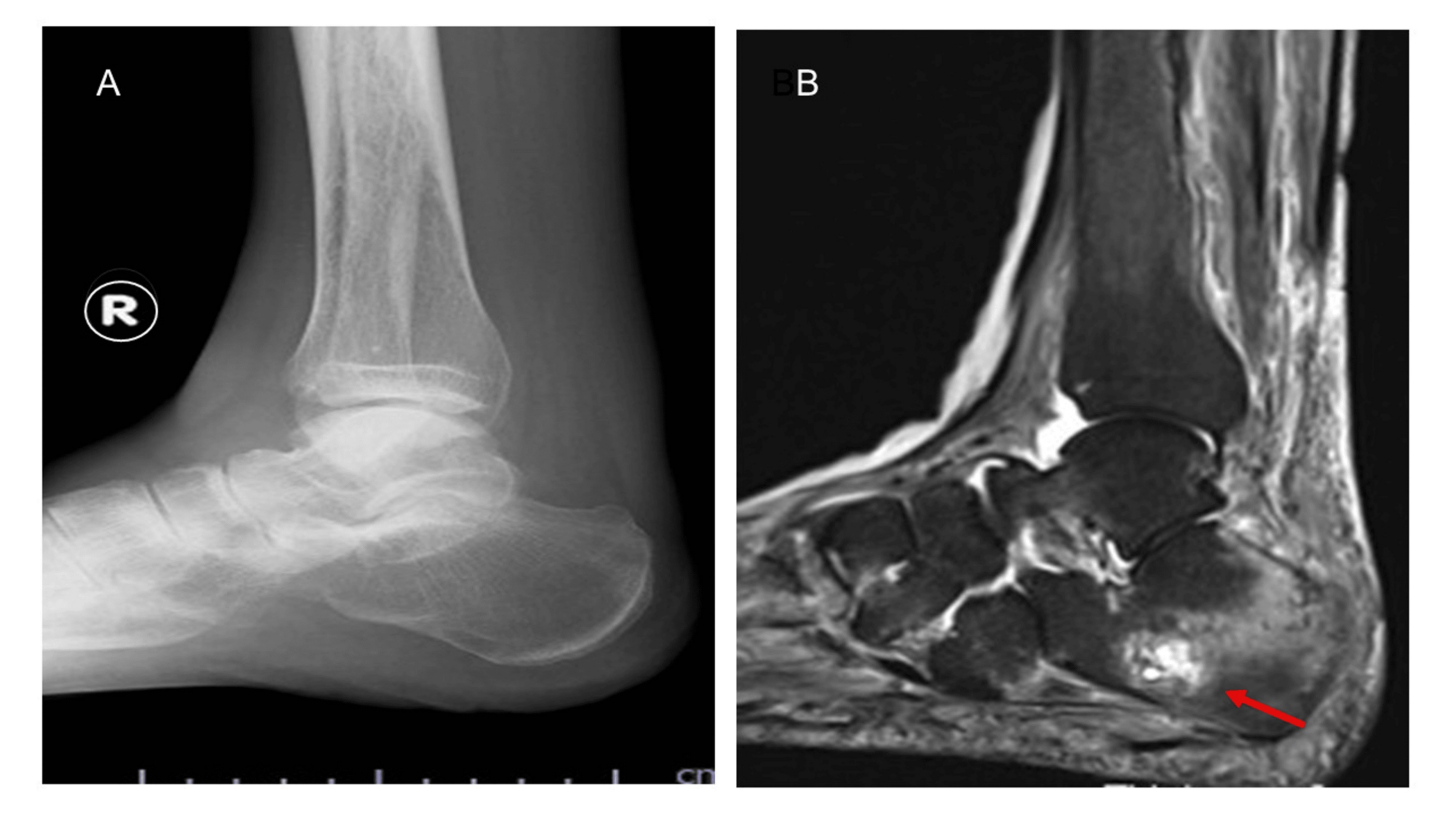
Imaging of the right calcaneus during the dialysis initiation period. (A) Plain radiograph shows no apparent abnormality. (B) Short tau inversion recovery (STIR) magnetic resonance imaging (MRI) demonstrates a high-signal intensity area at the posterior calcaneal trabeculae (arrow), consistent with bone marrow edema due to occult insufficiency fracture.

Plain radiographs and computed tomography (CT) showed no definite abnormalities. In contrast, MRI revealed multiple insufficiency fractures involving the right calcaneus, first and second right metatarsals, and left calcaneus (Figure 1). Bone mineral density (BMD) in the right calcaneus and one-third distal radius was reduced to 69% of the young adult mean (YAM) as assessed by the osteo-sono assessment index (OSI), and to 81% of the YAM as assessed by dual-energy X-ray absorptiometry (DXA), respectively.

Laboratory findings on admission were as follows: intact parathyroid hormone (PTH), 696 pg/mL; alkaline phosphatase, 64 U/L; serum zinc, 68 µg/dL; serum IgA, 180 mg/dL; and skeletal muscle mass index, 5.5 kg/m^2^ (Table 1).

**Table 1:** Laboratory data on admission ALP: alkaline phosphatase; TSH: thyroid-stimulating hormone; RBC: red blood cell; HPF: high power field; US: ultrasonography; BMI: body mass index; SMI: skeletal mass index; DXA: dual-energy X-ray absorptiometry; OSI: osteo-sono assessment index; ABI: ankle-brachial index

Parameter	Result	Reference range
Albumin	3.5	3.8–5.3 g/dL
Corrected calcium	7.8	8.4–10.0 mg/dL
Phosphate	3.6	3.5–6.0 mg/dL
Magnesium	1.7	1.8–2.5 mg/dL
Intact PTH	696	60–240 pg/mL
ALP	64	38–113 U/L
Zinc	68	80–130 µg/dL
Osteocalcin	57.1	8.3–32.7 ng/mL
Free carnitine	44.4	36–74 µmol/L
Homocysteine	82.4	5–15 nmol/mL
Vitamin B12	163	180–914 pg/mL
Folic acid	4.0	> 4.0 ng/mL
Free T3	< 1.50	1.71–3.71 pg/mL
Free T4	0.88	0.70–3.71 ng/dL
TSH	1.87	0.35–4.94 µIU/mL
IgA	180	110–410 mg/dL
Urinalysis:		
Protein	26.8	< 15 mg/dL
RBC	0–1	< 5 /HPF
BMI	15.8	18.5–25.0 kg/m2
SMI	5.5	> 7.0 kg/m2
Parathyroid size (US)	6 × 6 × 5	< 3 × 3 × 3 mm
Kidney (US: long axis)	< 8	10 – 12 mm
ABI	1.20 / 1.20	0.91–1.40
Radius DXA	81	> 80% YAM
Calcaneus OSI	69	> 80% YAM

**Figure 2:**
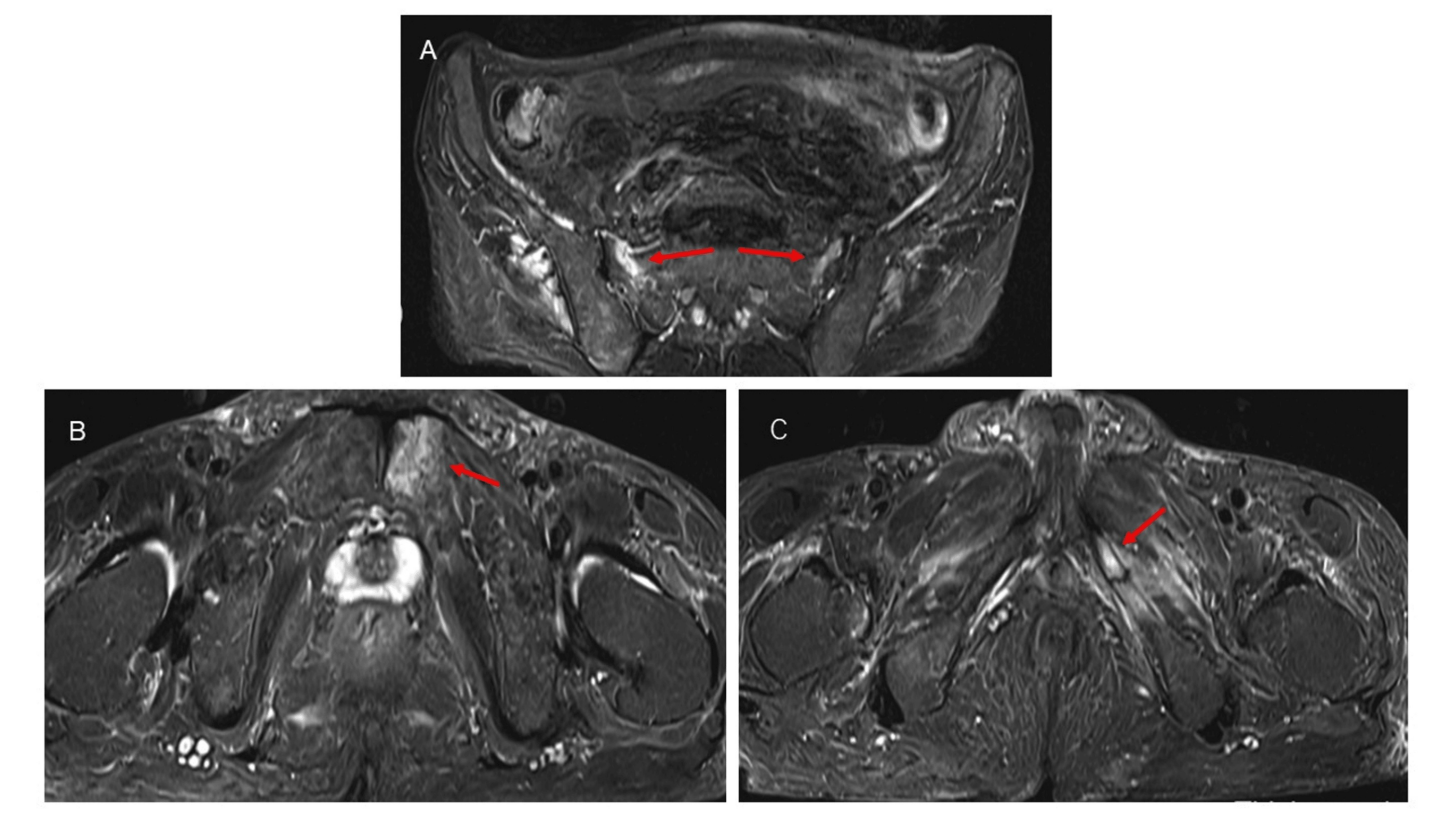
Pelvic insufficiency fractures detected after hemodialysis initiation. (A–C) Axial Short tau inversion recovery (STIR) magnetic resonance imaging (MRI) reveals high-signal intensity areas at the bilateral sacrum (A), left pubis (B), and left ischium (C) (arrows), consistent with bone marrow edema due to insufficiency fractures undetectable on CT.

**Figure 3:**
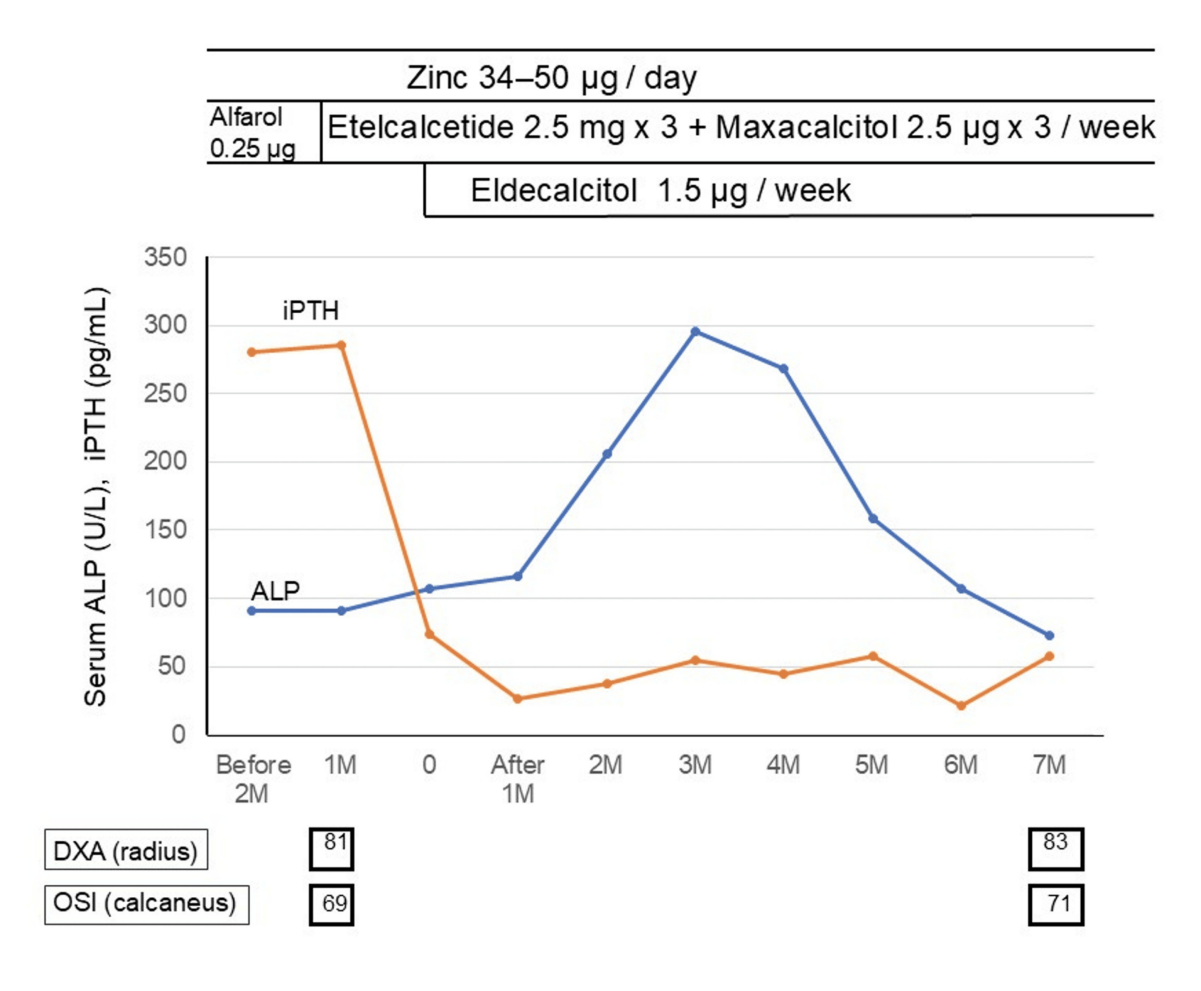
Changes in serum alkaline phosphatase (ALP), intact parathyroid hormone (iPTH) levels and bone mineral density after initiation of dialysis and zinc supplementation. Serum ALP (blue line) and iPTH (red line) were monitored before and after dialysis initiation. Zinc (34-50 µg/day), etelcalcetide (2.5 mg × 3/week) + maxacalcitol (2.5 µg × 3/week), and eldecalcitol (1.5 µg/week) were administered as shown in the figure. Bone mineral density by both dual-energy X-ray absorptiometry (DXA, radius) and osteo-sono assessment index (OSI, calcaneus) slightly increased during follow-up.

Given the fracture burden and biochemical profile, alfacalcidol was discontinued and replaced with intravenous etelcalcetide and maxacalcitol. One month after the drug switch, the patient developed lower back pain, and an MRI demonstrated additional insufficiency fractures of the bilateral sacrum, left pubis, and ischium, without vertebral compression or femoral neck fractures (Figure 2). Consequently, eldecalcitol and oral zinc supplementation were introduced, and the patient’s OSI and DXA increased to 71% and 83% of the YAM, respectively, after seven months of treatment (Figure 3). During the subsequent 17 months of hemodialysis, the patient’s bone pain gradually resolved, and no new fractures were detected.

## Discussion

This case highlights two clinically important aspects in long-term CKD management: (1) the occurrence of multiple occult fractures at the transition to dialysis that were detectable only by MRI, and (2) the potential contribution of zinc deficiency to bone fragility in the CKD-MBD context.

Occult fractures at the initiation of dialysis

Our patient developed multiple occult fractures, including bilateral sacral, pubic, ischial, calcaneal, and metatarsal fractures, within two months of hemodialysis initiation, 14 years after his serum creatinine first exceeded 3.0 mg/dL. MRI was performed selectively in symptomatic regions when localized pain persisted despite negative radiographic and CT findings. Initially, a foot MRI (ankle to metatarsal level) was obtained for ankle and dorsal foot pain, followed by a pelvic MRI when new lower pain developed. MRI provided the definitive diagnosis, consistent with its superior sensitivity in detecting pelvic and stress fractures [3]. As pelvic fragility fractures are often underdiagnosed due to nonspecific symptoms and the poor sensitivity of conventional imaging, MRI is particularly valuable for diagnosis when a fracture is suspected despite negative radiographs. Brown pseudotumors of bone related to secondary hyperparathyroidism were considered in the differential diagnosis; however, the absence of expansile osteolytic lesions and the MRI pattern dominated by diffuse marrow edema favored the diagnosis of insufficiency fractures rather than pseudotumor. Whole-body MRI screening may be potentially useful in dialysis patients presenting with unexplained pain or functional decline, although the cost-effectiveness and optimal screening strategy remain to be elucidated.

IgA nephropathy progresses slowly but may ultimately culminate in ESKD after decades. Meanwhile, CKD-MBD can begin in earlier CKD stages and may remain unrecognized until overt skeletal events occur. Fracture risk is known to rise as kidney function declines, with the highest rates, particularly for hip and non-vertebral fractures, observed in stage 5 CKD and patients undergoing dialysis [4]. Epidemiological studies indicate that the incidence of hip fractures increases sharply during the three months before dialysis initiation, peaks at dialysis initiation, and declines thereafter. In contrast, the incidence of non-hip fractures, including pelvic fractures, rises more gradually after dialysis initiation [5]. These temporal patterns may reflect acute disturbances in bone turnover, mineral metabolism, and mobility during the transition to dialysis. Our case highlights that occult pelvic fractures may represent a hidden but clinically important component of this high-risk period.

Zinc deficiency and bone fragility

The second clinically relevant aspect of this case was the coexistence of hypozincemia and reduced calcaneal BMD (69% YAM) at the time of fracture diagnosis. Although OSI and DXA values help describe bone status, the clinical interpretation of OSI% YAM in dialysis patients has not been evaluated.

Zinc deficiency is common in CKD and is driven by reduced dietary intake, impaired absorption, adsorption by phosphate binders, urinary and dialytic losses, and increased utilization under oxidative stress [6]. Medications frequently prescribed in CKD, such as diuretics, phosphate binders, and iron preparations, may further exacerbate zinc depletion [7, 8].

Notably, zinc plays multiple roles in skeletal biology, serving as a structural component of bone and a cofactor for numerous enzymes involved in bone metabolism [9]. At the cellular level, zinc stimulates osteoblast differentiation, inhibits osteoclast activity, regulates collagen apatite alignment, and modulates vitamin D receptor signaling [10, 11]. Approximately 30% of total body zinc is stored in the bone, and reduced cancellous bone zinc concentrations have been observed in osteoporosis [12].

Furthermore, zinc deficiency may attenuate the therapeutic efficacy of antiresorptive and anabolic osteoporosis therapies such as denosumab and eldecalcitol [13].

Clinical data suggest that zinc supplementation improves BMD and may prevent fractures in older patients with osteoporosis and zinc deficiency [14]. Only a few studies have addressed zinc and bone health in patients undergoing hemodialysis; however, zinc supplementation has been shown to increase zinc and bone-specific alkaline phosphatase, suggesting enhanced bone formation [15]. Our case adds clinical support to the concept that zinc deficiency contributes to skeletal fragility in CKD-MBD.

Therapeutic implications

Current evidence guiding osteoporosis management in advanced CKD (stages 4-5 and dialysis) remains limited, and consensus guidelines cannot provide definitive recommendations [16]. In our patient, the combination of calcimimetics (etelcalcetide), active vitamin D analogs (maxacalcitol and eldecalcitol), and zinc supplementation was associated with gradual symptom resolution and no new fractures during a 17-month follow-up period. Calcimimetics suppress PTH secretion and may exert direct anabolic effects on bone through calcium-sensing receptor signaling in osteoblasts and osteoclasts [17]. Lower PTH levels in dialysis patients are associated with reduced fracture risk [18], and calcimimetic therapy has been linked to improvements in bone histology and a lower fracture incidence [19].

Eldecalcitol, a vitamin D analog with both antiresorptive and mineralizing-based actions, further improves bone quality and reduces fracture risk [20].

In our case, zinc supplementation may represent an underutilized adjunct, correcting a prevalent micronutrient deficiency that impairs bone quality and possibly blunts the efficacy of established therapies.

## Conclusions

Our case suggests that multiple occult fractures in patients with chronic renal insufficiency can arise insidiously during the transition to dialysis, even in the absence of overt trauma, and may only be identified using advanced imaging modalities such as MRI. In patients with CKD, particularly those with secondary hyperparathyroidism and micronutrient deficiencies such as hypozincemia, fracture risk reflects reduced bone mass as well as impaired bone quality.

Early skeletal assessment, proactive application of sensitive diagnostic tools, and comprehensive correction of metabolic and nutritional disturbances, including zinc supplementation, should be considered as essential components of CKD-MBD management. Such strategies may enable timely detection, support fracture healing, and ultimately improve long-term musculoskeletal outcomes in this high-risk population.
